# “Emotion”: The History of a Keyword in Crisis

**DOI:** 10.1177/1754073912445814

**Published:** 2012-10

**Authors:** Thomas Dixon

**Affiliations:** School of History, Queen Mary, University of London, UK

**Keywords:** affection, emotion, definitions, history, passion, semantics

## Abstract

The word “emotion” has named a psychological category and a subject for systematic enquiry only since the 19th century. Before then, relevant mental states were categorised variously as “appetites,” “passions,” “affections,” or “sentiments.” The word “emotion” has existed in English since the 17th century, originating as a translation of the French *émotion*, meaning a physical disturbance. It came into much wider use in 18th-century English, often to refer to mental experiences, becoming a fully fledged theoretical term in the following century, especially through the influence of two Scottish philosopher-physicians, Thomas Brown and Charles Bell. This article relates this intellectual and semantic history to contemporary debates about the usefulness and meaning of “emotion” as a scientific term.

“Emotion” has, since 1884, been a theoretical keyword at the heart of modern psychology. In that year William James wrote an influential article in *Mind* entitled “What Is an Emotion?” A century and a quarter later, however, there seems to be little scientific consensus on the answer to his question, and some are beginning to wonder whether it is the very category of “emotion” that is the problem.

Izard’s (2010a) interviews with leading emotion scientists, together with responses from other experts, powerfully demonstrate that, despite the continuing proliferation of books, journals, conferences, and theories on the subject of “emotion,” there is still no consensus on the meaning of this term. Some even believe that it should be thrown out of psychology altogether. Among the scientists surveyed by Izard, there was moderate support for the view that the term “emotion” is “ambiguous and has no status in science,” and that it should therefore be abandoned (2010a, pp. 367–368). “Emotion” is certainly a keyword in modern psychology, but it is a keyword in crisis. Indeed, as I shall suggest below, it has been in crisis, from a definitional and conceptual point of view, ever since its adoption as a psychological category in the 19th century.

Izard’s recent article and several of the responses to it (White, 2010; Widen & Russell, 2010; Wierzbicka, 2010) ask questions about the language of “emotion”: whether it forms part of a universal human “folk psychology,” whether it is part of “ordinary language” in English, and whether, in light of the answers to these questions, it can be expected to operate as part of a truly scientific lexicon. An historical perspective can help to answer these questions.

Historians have long recognised the importance of keywords as both mirrors and motors of social and intellectual change (Dixon, 2008; Williams, 1976). This is especially true in the realms of culture and thought, where new words, or new meanings attached to old ones, can create new concepts, and even new worldviews, which in turn transform people’s ability to imagine, experience, and understand themselves. Psychological categories and concepts in particular have this reflexive relationship with our mental lives, shaping and colouring as well as explaining them (Khalidi, 2010; R. Smith, 2005, 2007). The history of the term “emotion” as a keyword of just this kind is both shorter and more eventful than its modern users might imagine. Although the word “emotion” (imported into English from the French *émotion*) was in use in the 17th and 18th centuries, it did not become established as the name for a category of mental states that might be systematically studied until the mid-19th century. The present article uses the intellectual history of this term to offer an historical diagnosis of the contemporary definitional malaise, and to offer a reminder of some of the ideas about passions, affections, and emotions that have been forgotten during the last two centuries.

As Izard (2010b) rightly points out about the current debates, the problem is not that the term “emotion” has no clear meaning, but that it has many meanings (2010b, p. 385). This has been the case historically too. I have divided this article into three sections which correspond to three different dimensions of those multiple meanings: categories, concepts, and connotations. By thinking about categories, we can investigate which mental states have been thought to fall into the category of “emotion,” and what alternative mental typologies have been used, especially those which made a fundamental distinction between “passions” and “affections.” Secondly, by looking at the multiple concepts that have been named by the single term “emotion,” we can ask what theorists have intended to claim about a mental or bodily state by calling it an “emotion.” From the outset, there was ambiguity and confusion. Finally, in the realm of connotations, we have access to those broader intellectual, linguistic, and disciplinary frameworks within which keywords function. We will see that the different cultural territories within which the words “passions” and “emotions” operated gave them different roles in the production of both mental experiences and of psychological theories. These reflections on connotation will pave the way for some brief concluding thoughts on “emotion” as a term in both everyday and scientific language in the 21st century, and the morals we can draw from history.

## Categories

The first books written on the subject of “the emotions” appeared between the 1830s and 1850s (Bain, 1859; Cooke, 1838; Lyall, 1855; Ramsay, 1848). Until then, philosophers, physicians, moralists, and theologians generally used more than one term with which to theorise about mental states which would later be designated “emotions.” Theorists distinguished especially between “passions” on the one hand and “affections” on the other. In 1836 the English polymath William Whewell commented that the proposal to refer to what he called “the desires and affections” of human nature as “the Emotions” had not been generally accepted. Even as late as 1862, Whewell was expressing his preference for the compound phrase “the desires and affections,” while acknowledging that the term “emotional” had been adopted by some recent writers (Whewell footnotes to Mackintosh, 1862, pp. xlv, 79; see also Dixon, 2003, pp. 186–187).

In order to understand this all-important distinction between troubling desires and passions on the one hand and milder affections and sentiments on the other, we need to look back briefly to ancient debates between Stoicism and Christianity. An analysis of works by two of the most influential medieval Christian theologians, Augustine of Hippo and Thomas Aquinas, reveals that it was their desire to provide an alternative to the moral philosophy of the Greek and Roman Stoics that led to their creation of the distinction between passions and affections (Dixon, 2003). The Stoics had famously treated all the passions as diseases of the soul, from which the wise man could be cured by the application of calm reason. When the Stoic sage felt the “first movements” of a passion stirring with him, he was advised to withhold his assent from the judgement underlying that incipient passion, thus retaining his composure and peace of mind, his *apatheia*. The Stoics aimed thus to use a kind of cognitive therapy to remain free of passions and perturbations of the mind, while still being able to enjoy milder positive feelings known as *eupatheiai* (Annas, 1992; Sorabji, 2000).

The response of Augustine and Aquinas to this Stoic view was twofold. In one way, they agreed with the Stoics: The passions were indeed violent forces that could conflict with reason and lead an individual into sin. But, on the other hand, they did not agree that a state of complete Stoic *apatheia* was one to be wished for. As Augustine put it, someone who no longer trembled from fear or suffered from sorrow would not have won true peace, but would rather have lost all humanity (Augustine, 1966, XIV.9). It was important for theologians to be able to distinguish between those troubling movements of the soul—appetites, lusts, desires, passions—that the good Christian should avoid, and those more virtuous and Godly affections of love and compassion to which they might rightly aspire. For Aquinas, the passions and affections were movements of two different parts of the soul, namely the sense appetite and the intellectual appetite respectively. The latter was another term for the will.

This distinction between passions of the sense appetite and affections of the intellectual appetite, although interpreted variously by different theorists and only rarely elaborated in detail, undergirded moral-philosophical thought for many centuries. The distinction was explicitly discussed in several philosophical works (e.g., Charleton, 1701; Hutcheson, 1728/1742). A treatise about religious affections by the American preacher and philosopher Jonathan Edwards emphasised that affections were movements of the intellectual part of the soul:

Holy affections are not heat without light, but evermore arise from some information of the understanding, some spiritual instruction that the mind receives, some light or actual knowledge. (Edwards 1746/1959, p. 266)

The 18th century saw a proliferation of new ideas about sentiments and sensibility, as well as about passions and affections. But in almost all theoretical works, the various feelings and emotions of the human heart and intellect were understood to fall into at least two categories: the more violent and self-regarding “passions” and “appetites” on the one hand, and the milder and more enlightened “interests,” social “affections,” and “moral sentiments” on the other (DeJean, 1997; Dixon, 2003; Hirschman, 1997). A multivolume work on the passions and affections of the mind composed in the early 19th century by the physician and philosopher Thomas Cogan restated the semantic distinction between passions and affections, noting that in common usage the word “passion” was often applied to “the *evil* propensities,” while “affection” was used for the “*virtuous* propensities; as the *social, friendly, parental, filial* affections” (1802, p. 3). And, as we have already seen, as late as 1862 Whewell was expressing his preference for the compound phrase “the desires and affections.”

This more differentiated typology was lost with the rise of the capacious new category of “emotion” during the 19th century. The key figure in this transition was the Edinburgh professor of moral philosophy Thomas Brown, whom I have previously designated the “inventor of the emotions” (Dixon, 2003, p. 109). Brown subsumed the “appetites,” “passions,” and “affections” under a single category: the “emotions.” The word “emotion” was already in wide usage, but in Brown’s lectures, first published in 1820, the term took on a newly systematic theoretical role in the science of the mind. This innovation proved to be popular. In arguably the first modern psychological book about the emotions, the incredibly wide reach of the new category was made explicit: “Emotion is the name here used to comprehend all that is understood by feelings, states of feeling, pleasures, pains, passions, sentiments, affections” (Bain, 1859, p. 3). Two decades later, McCosh (1880) enumerated over 100 discrete feeling states that fell into the category. How could anyone possibly devise a single theory, or a simple conceptual definition, that could cover such a wide range of different mental states? The answer is that no one could.

## Concepts

The word “emotion” first arrived on British shores from France in the early 17th century. John Florio, the translator of Michel de Montaigne’s celebrated essays, apologised to his readers for the introduction of various “uncouth termes” from French into his English version of the work, including among them the word “emotion” (de Montaigne, 1603, p. v). In both its French and English forms, “emotion” was a word denoting physical disturbance and bodily movement. It could mean a commotion among a group of people (as in the phrase “public emotion”), or a physical agitation of anything at all, from the weather, or a tree, to the human body (DeJean, 1997; Diller, 2010).

Increasingly, during the 18th century, “emotion” came to refer to the bodily stirrings accompanying mental feelings. The Stoic idea of “first movements,” those physical stirrings that marked the onset of a passion, was sometimes referred to with the phrase “first emotions of passion,” as it was in a sermon preached before the queen of England in 1711 on the subject of “The government of passion” and also in Fielding’s 1749 novel *The History of Tom Jones* (Clarke, 1738, p. 426; Fielding, 1749, Vol. 2, p. 306). And for some medical and philosophical writers, the term “emotion” was reserved for those bodily movements which served as the external signs of inward passions and affections. Bentham (1789/1996) wrote that “The emotions of the body are received, and with reason, as probable indications of the temperature of the mind” (1789/1966, p. 63; cf. LeBrun, 1734, pp. 21, 34). This usage was continued into the early 19th century by Cogan (1802), who insisted that the term “emotions” was properly applicable only to those “sensible changes and visible effects which particular passions produce upon the frame” (1802, pp. 7–8). This idea, that emotions were external and visible effects, also explains why the term “sensible” (meaning outwardly observable) was so frequently applied to the term “emotion” in 18th-century texts (Diller, 2010, p. 150).

Finally, from the mid-18th century onwards, “emotion” moved from the bodily to the mental domain. As early as 1649, Descartes had attempted to introduce the term *émotion* as an alternative to *passion* in his theoretical treatise on the passions of the soul (DeJean, 1997; S. James, 1997). His suggestion was not generally followed, however, and the earliest works to make frequent use of “emotion” as a term for feeling, passion, and related states of mind did not appear until about 100 years later, in the 1740s and 1750s. These included important philosophical works by two central figures of the Scottish Enlightenment, Hume (1739–1740) and A. Smith (1759). Their uses of the term were far from systematic, however. For them, as for many other writers in the second half of the 18th century, “emotion” functioned either as an undefined and general term for any kind of mental feeling or agitation, or sometimes as a stylistic variant for central theoretical terms such as “passion” and “affection” (Dixon, 2003).

As I have already indicated, it was in the early 19th-century lectures of another Scottish philosopher, Thomas Brown, that the term “emotion” definitively took on its new status as a theoretical category in mental science, replacing those “active powers” of the mind, the “passions” and “affections.” Brown, a physician and poet as well as a philosopher, was the first to treat “emotion” as a major theoretical category in the academic study of the mind, and his use was the most systematic and most influential of the period. Here, then, in the lecture halls of Edinburgh University in the years between 1810 and 1820, we arrive at the key moment in the history of our modern concepts of “emotion.”

What, then, was the definition that Professor Brown ascribed to this important new theoretical term in mental science? “The exact meaning of the term *emotion*,” Brown told his students, “it is difficult to state in any form of words.” And it has remained so ever since. Brown did go a little further than this in trying to offer a definition of the “emotions”:

Perhaps, if any definition of them be possible, they may be defined to be vivid feelings, arising immediately from the consideration of objects, perceived, or remembered, or imagined, or from other prior emotions. (Brown, 1820/2010, pp. 145–146)

In other words, unlike sensations, which were caused directly by external objects, emotions were caused by the mental “consideration” of perceived objects; and, unlike intellectual states, they were defined as noncognitive “vivid feelings” rather than as forms of thought.

Brown’s lectures exercised a very wide influence in the decades between 1820 and 1860, and it became standard to repeat his statement that the term “emotion” was difficult to define except in terms of vividness of feeling. Although everyone apparently knew what an “emotion” was, theorists agreed with Brown that this could not be embodied in any verbal definition (Dixon, 2003, pp. 129–130). Two hundred years later, we are still living with this legacy of Thomas Brown’s concept of “emotion.” Psychologists have continued to complain, at regular intervals, right up to the present, that “emotion” is utterly resistant to definitional efforts (Izard, 2010a, 2010b). This is hardly surprising for a term that, from the outset, was defined as being indefinable.

Brown’s was also a strongly noncognitive concept of “emotion.” His stark separation between intellectual thoughts and emotional feelings was endorsed by many of the leading psychologists of the late 19th century. Bain (1855, 1859), McCosh (1886, 1887), Baldwin (1891), and Sully (1892) all produced two-volume textbooks of psychology in which Volume 1 was devoted to the senses and the intellect, and Volume 2 to the emotions, feelings, and will.

But Brown was not the only important early “emotion” theorist, and so his is not the only relevant legacy. A second key figure was another Edinburgh physician and philosopher, Charles Bell. Bell was an important figure in the history of neurology and also the most influential 19th-century theorist of expression before 1872, when Darwin published his work on the subject. Bell’s theories of emotion and expression, worked out in the successive editions of his essays on the anatomy and philosophy of expression published between 1806 and 1844, provided foundations later built upon by both Darwin and James. It is well known that Darwin argued strongly against the theological notion in Bell’s work that the muscles of the human face had been divinely designed to express the higher sentiments. What has only rarely been noticed, however, is that Darwin took his main theoretical principle of expression, namely the idea of “serviceable associated habits,” directly from Bell’s work (Darwin, 1872; Dixon, 2003).

Such is Bell’s importance, in fact, in this conceptual history that it would be appropriate to think of him as the coinventor of the modern “emotions” along with Brown. Where Brown was the key theorist of “emotions” as vivid mental feelings with mental causes, in Bell’s work we find a concept of “emotion” which for the first time gave a constitutive role to bodily movements.

For Bell an “emotion” was a movement of the mind. His brief definition of the term was that “emotions” were “certain changes or affections of the mind, as grief, joy, or astonishment,” which could become visible through “outward signs” on the face or body (Bell, 1824, p. 19). The additional interest of Bell’s work, however, is the importance he gave to bodily movements, especially of the heart and lungs, as not only outward signs, but also as constitutive causes of emotional experience. He recognised that the idea that the emotions might “proceed from or in any degree pertain to the body” might not “willingly be admitted” by his readers (Bell, 1824, pp. 20–21). Nonetheless, he tried to persuade them that the “organs of breathing and speech” were necessary not only to the “expression” of emotions, but also to their “development.” Bell pressed the point further, arguing that the operation of the organs of expression preceded “the mental emotions with which they are to be joined,” and strengthened and directed them. He even argued that the reason that all people experienced the same “internal feelings and emotions or passions” was because of the uniform operation of the bodily organs (Bell, 1824, pp. 20–21). The parallel here with W. James’s famous formulation, published six decades later, is very striking. W. James was certainly familiar with Bell’s work, although only making a passing reference to him in his 1884 article (p. 191). Darwin (1872) had also endorsed a similar view of the indispensable role of bodily movements in a fully fledged emotion, suggesting that: “Most of our emotions are so closely connected with their expression, that they hardly exist if the body remains passive” (1872, pp. 239–240).

In Bell’s works, then, we find the final piece of the jigsaw. Here was the source of the idea that the term “emotion” referred to mental states that necessarily had an outward bodily expression, which additionally somehow constituted the emotion. Taken in combination with Brown’s influential treatment of the “emotions” as a very broad category of noncognitive states of feeling, we now have a clear picture of the origins of the late 19th-century theories of emotion which have given rise to so many conceptual and definitional problems. While Brown and Bell agreed that an “emotion” was itself something mental, they differed over whether its constituents were primarily mental or bodily. The tensions between these two models were never fully resolved. Darwin and James were both influenced by these works produced in Edinburgh in the opening 2 decades of the 19th century. Darwin even studied medicine in Edinburgh briefly in the 1820s, and James stated that he spent his youth immersed in philosophical works by Brown and by Brown’s predecessor in the moral philosophy chair, Dugald Stewart (W. James, 1902/1985, p. 2). For centuries, theorists have debated what should be considered the true seat of the emotions: the soul or the body; the heart or the brain (Bound Alberti, 2010). In view of the importance of Brown and Bell in this conceptual history, I would suggest that the true seat of the “emotions” was in fact the University of Edinburgh, circa 1820 (Dixon, 2006).

## Connotations

It is appropriate that at this stage of the argument we should have reached a conclusion about a particular institution and a particular place. This reminds us that words do not operate in vacuums, but rather within lexical and social networks. Words derive their meanings from the company they keep, and that applies both to the other words they rub shoulders with, and to the speakers, writers, and readers through whose minds, mouths, hands, and eyes they pass.

The semantic connotations that were lost by the transition from “passions” and “affections” to “emotions” in theories of the human mind can all be grouped together under the unifying theme of pathology: cognitive, medical, or moral (Dixon, 2006, 2011). “Passion” and “affection” were both terms whose etymology and core meanings emphasised passivity, suffering, and disease. For the Stoics, the passions had been diseases of the soul, to be cured by cognitive therapy, and right up to the 19th century the terms “passion” and “affection” were used in medical contexts as terms for organic disease. Passions and affections of the mind, especially in their stronger forms, were also considered by physicians to constitute a constant threat to health and life. Since the key early “emotion” theorists, including Brown and Bell, were almost all trained medics, it is significant that they chose to use a word for the vivid mental feelings which detached them from this medical thought-world and its pathological associations, bestowing to subsequent generations of mental philosophers and psychologists a newly de-medicalised concept (Dixon, 2006).

Questions of professional and disciplinary identity are also germane to thinking about the detachment of “emotion” from the established languages of morality and religion. Many of the most influential theorists of “passions” and “affections” had been moral philosophers, clergymen, or both. Preachers and theologians, as well as secular moralists, most often found themselves discoursing on these subjects in order to demonstrate the importance of governing the passions and cultivating the affections. During the religious revivals of the 18th century, in which the feelings and sympathies of the human heart were so important, preachers such as Edwards (1746/1959) and Whitefield (1772) spoke and wrote in the language of the Bible. The terms “passions,” “lusts,” “desires,” and “affections” all had a biblical pedigree. The term “passion” had an additional biblical association through its connection with the gospel accounts of the sufferings and death of Jesus of Nazareth. The terms “emotion” and “emotions,” by contrast, were detached from the linguistic worlds of theology and moralism. They never appeared in any English translation of the Bible and, unlike the terms “passion” and “passions,” were very unlikely to be paired with such moralising epithets as “despicable,” “detestable,” “evil,” “perverted,” or “vicious” (Diller, 2010, p. 150; Dixon, 2011).

When modern uses of “emotion” and “emotional” emerged during the 19th century, they connoted knowledge of and sympathy with a modern and scientific approach to human mental life. They were words which belonged within a secular, morally neutral, and scientific register. The linguistic shift from “passions” and “affections” to “emotions” thus both reflected and enabled shifts in institutional and intellectual authority. By the end of the 19th century the view was on the rise in European and American universities that a properly scientific account of the human mind would be produced only through a thoroughly physiological investigation. Champions of this view explicitly contrasted their work with the philosophical psychology of their predecessors (and contemporaries), which they believed was still in thrall to theological, spiritual, and dualistic views of the human person.

One particularly able critic of the physiological tendency of modern psychology in general, and of James’s theory of emotion in particular, was David Irons. Irons argued in several articles in the 1890s and in his book on moral psychology (1903) that emotions were irreducible “attitudes” of the whole person (Dixon, 2003; Gendron & Barrett, 2009, p. 325). It is notable that Havelock Ellis’s response to Irons attacked him not only for his theoretical views, but for his ignorance of human physiology and his reliance on the tools of philosophy. Ellis wrote that the problems of modern psychology required “something more than a merely logical equipment; they require a very considerable physiological and even pathological equipment” and that anyone who could suggest, for instance, that melancholia lacked a physical basis was evidently “not competent to discuss the nature of emotion” (Ellis, 1895, p. 160).

In summary, the term “emotion” suited the purposes of a self-consciously secularising and scientific cadre of psychological theorists in the late 19th century, detached as it was from the centuries of moral and theological connotations that had accrued to the terms “passion” and “affection.” “Emotion,” for progressives such as Ellis, was the name of a domain of scientific study from which mere philosophers were to be barred. Competence to discuss emotion now required a training in physiology. As W. James put it at the end of his 1884 *Mind* article, the truth or falsity of his theory of emotion would be best determined not by logical analysis, but by empirical investigations undertaken by “asylum-physicians and nervous specialists” who “alone have the data in their hands” (1884, p. 204).

## Conclusions

So, when W. James famously asked in 1884, “What is an emotion?” he was not engaging with an age-old conundrum, but was seeking to define a psychological category that had been in existence only a couple of generations. James’s answer to his own question, one which revealed his indebtedness to Brown, Bell, and Darwin, was that emotions were vivid mental feelings of visceral changes brought about directly by the perception of some object in the world.

James’s theory had a curious early career. On the one hand, it became, along with the similar theory of the Danish psychologist Carl F. Lange, the flagship emotion theory of the fledgling science of psychology. On the other hand, the theory entirely failed to create consensus among the psychological community except, perhaps, a consensus that it was wrong. Within 10 years of the publication of James’s original theory it had been systematically rebutted in almost all the leading philosophical and psychological journals. Critics in the 1880s and 1890s argued that James’s theory failed to distinguish between emotions and nonemotions; that it failed to differentiate between the different emotions; that it gave excessive priority to feelings of bodily change at the expense of other components of emotion; and that it unnecessarily denied the role played by cognitive and intellectual factors in generating emotion. James’s article created further confusion by seeming to support several different claims about whether all emotions had bodily expressions, or only some, and whether this was a matter of definition, or of empirical discovery (Dixon, 2003; Ellsworth, 1994; Feinstein, 1970).

After ten years of rebuttals of his original theory, W. James duly published (1894) a restatement of his views on emotion, which included so many concessions and qualifications as to amount virtually to a retraction of his own theory. So, by the 1890s, although the idea that “emotion” was the name of a psychological category had become entrenched, the nascent psychological community had neither an agreed definition of the extent of the category, nor a shared idea of the fundamental characteristics of the states that fell within it.

The founders of the discipline of psychology in the late 19th century bequeathed to their successors a usage of “emotion” in which the relationship between mind and body and between thought and feeling were confused and unresolved, and which named a category of feelings and behaviours so broad as to cover almost all of human mental life including, as Bain (1859) had put it, all that was previously understood by the terms “feelings, states of feeling, pleasures, pains, passions, sentiments, affections” (1859, p. 3).

The survey undertaken recently by Izard (2010a) reveals that psychologists are still living with this legacy. On the basis of the replies to his questionnaire, Izard put together a composite description of what contemporary emotion scientists mean by “emotion.” The most commonly cited features were summarised by Izard in one sentence:

Emotion consists of neural circuits (that are at least partially dedicated), response systems, and a feeling state/process that motivates and organizes cognition and action. (2010a, p. 367)

Izard emphasised that this was not meant to be a definition of “emotion,” but a description of the dominant uses. Nonetheless, it indicates well enough the challenges that still face theorists of “emotion,” especially the need somehow to articulate the assumed relationships between physiological processes and mental experiences, and between states of feeling and states of thought. Among those philosophical and psychological writers of the 19th century (and before) whose works have been excluded from the canon of the history of psychology, but who resisted the conglomeration of “passions” and “affections” into “emotions,” who argued for the centrality of the intellect and cognition to states of feeling, and who connected psychology most closely to philosophy and ethics rather than to physiology, some clues may still be found as to what went wrong in the construction of modern concepts of “emotion” in psychology (Dixon, 2003, 2011; Gendron & Barrett, 2009).

In the debate about whether “emotion” can today function as a scientific term, its semantic and conceptual history is, it seems to me, relevant if not decisive. On the question of whether “emotion” is a “folk” or “everyday” term rather than a scientific one, there is a clear historical story to tell. “Emotion” has existed as a normal English-language term for physical agitation since the 17th century. It gradually started to be applied, in an undefined and general way, to states of mental feeling during the 18th century. So, when Brown and others adopted it as a term of mental science, they were adopting an everyday term and giving it a new theoretical role. As Brown himself put it: “Every person understands what is meant by an emotion” (1820/2010, p. 145). In the course of the 19th and 20th centuries, this previously “everyday” word became a scientific term used in technical ways not only in psychology, but also in medicine, sociology, and anthropology. The final stage of this semantic history has been the popularisation of ideas about “emotional intelligence,” “emotional literacy,” and “emotional well-being,” which have themselves virtually become “everyday” phrases. This process has seen scientific ideas about “emotion” and the “emotional” becoming widely spread through popular culture and politics, in a way that has traded on the scientific, psychological, and medical authority of that language.

This complex cultural history of “emotion,” especially its rather recent, haphazard, contested, and gradual emergence as an English-language psychological category in the first half of the 19th century, does not strongly suggest that “emotion” is likely to name either a natural kind or any kind of innate or “folk” psychological concept (cf. Barrett, 2006; Rorty, 2004; Wierzbicka, 2010). On the other hand, it may be that psychology is not the kind of science that deals in natural kinds or innate concepts. If the science of emotion is supposed to provide an explanation of a widely experienced kind of mental state, and in terms that can be communicated to the general public, then it might be better to stick with the complexity, fuzziness, and overinclusivity of “emotion” than to retreat still further from the world of everyday concerns into new scientific jargons.

Let me end, however, on a constructive note, by suggesting a third way between the retention of the problematic metacategory of “emotion” and its abandonment in favour of studies of discrete feeling states such as love, anger, fear, and the rest. In the conclusion of my 2003 book on this subject I was rather timid and suggested that the “old-fashioned terminology of passions and affections” was unlikely to “find favour in future psychological theories” (Dixon, 2003, p. 245). But perhaps now that the definitional crisis in “emotion” theories has reached a new peak, the time has come to reinstate in psychological science some version of that distinction between “passions” and “affections” which structured modern thought about mind and morality for so many centuries. Among philosophers of emotion, Griffiths (1997, 2003, 2004) in particular has lamented the overinclusivity of the modern category of “emotion” and argued that it should be divided into two subcategories: the more primitive “affect programs” and the “higher cognitive emotions” (cf. Elster, 1999; Rorty, 2004). If the lessons of history and philosophy are taken on board, then, it is just possible that the ideas of Augustine and Aquinas might yet turn out to be just what is needed to inspire a new scientific paradigm of emotions research for the 21st century.
